# Herbal, dietary supplements, oligo and trace elements consumption among chronic liver disease patients and caregivers: a cross-sectional study

**DOI:** 10.1007/s11739-026-04425-8

**Published:** 2026-06-23

**Authors:** Anna Licata, Stefano Ciriminna, Ottavia Tulone, Marcella Cammilleri, Laura Cilona, Roberta Chianetta, Lydia Giannitrapani, Roberto Citarrella, Mario Barbagallo, Maurizio Soresi

**Affiliations:** 1 Internal Medicine and Hepatology Unit, Department of Health Promotion, Mother and Child Care, Internal Medicine and Medical Specialties “G. D’Alessandro” (PROMISE), University of Palermo, Via del Vespro 141, Palermo, 90127 Italy; 2 Geriatric Unit, Department of Health Promotion, Mother and Child Care, Internal Medicine and Medical Specialties “G. D’Alessandro” (PROMISE), University of Palermo, Palermo, Italy; 3 Institute for Biomedical Research and Innovation (IRIB), National Research Council, 90146 Palermo, Italy

**Keywords:** Herbal dietary supplements, Oligo elements, Vitamins, Consumption, Liver diseases

## Abstract

Use of herbal and dietary supplements (HDS) is widespread among patients with chronic liver disease (CLD). Despite the potential for adverse effects (AEs) and drug interactions (ADRs), these products are often perceived as “natural,” safe, and not requiring medical prescription. This study aimed to assess HDS, oligo and trace element consumption among patients with CLD, to examine sociodemographic correlates, to determine the extent to which use is overseen by general practitioners or specialists versus self-medication, and to document reasons for use and perceived benefits. Over a 6-month period, patients with CLD attending the outpatient clinic of the Internal Medicine and Hepatology Unit of the University Hospital of Palermo, along with their caregivers, were surveyed regarding their use of supplements. A self-administered questionnaire collected information on demographics, lifestyle habits, supplement use, motivations, perceived benefits, and prescription awareness. A total of 323 participants were enrolled: 220 (68.1%) were CLD patients and 103 (31.9%) were caregivers. Overall, 110 (34%) subjects, 39 patients and 71 caregivers, reported using at least one supplement. Consumption was slightly more frequent among caregivers than patients (37.9% vs 32.3% *p* = ns). Among users, HDS were the predominant products (87/110; 79.1%), followed by oligo and trace elements (36/110; 33.0%); 21/110 (19.1%) reported combined use. The most common reasons for supplement use were anxiety and sleep disorders (each 15/110; 13.6%), osteoporosis/osteoarthrosis (15/110; 13,6%), dyslipidemia (12/110; 10.9%), and dyspepsia (11/110; 10.0%). Compared with caregivers, patients more frequently used supplements for anemia (7.3%), anxiety (13.6%), and steatotic liver disease (7.3%). Perceived benefit was reported by 70.9% of users, whereas AEs and ADRs were uncommon (3.6%). Supplement consumption for liver steatosis was more common among males than females (14.3% vs 2.9%; *p* = 0.034). Osteoporosis-related supplement use showed a significant positive correlation with age (*p* = 0.019). Consumption of HDS, oligo and trace elements is common among CLD patients, and it increases with age. Their use appears driven by perceived health knowledge and a desire for well-being. Further studies are needed to enhance health education and prevent inappropriate or excessive use of these products.

**Supplementary Information:**

The online version contains supplementary material available at https://doi.org/10.1007/s11739-026-04425-8.

## Introduction

Use of herbal and dietary supplements (HDS) is widespread and increases with age. In nationally representative US data (NHANES 2017–2018), 57.6% of adults reported using at least one supplement in the prior 30 days; use was consistently higher in women than men and rose till ~ 80% in women aged more than 60 years; multi-product use also rose with age, with a substantial proportion of adults reporting intake of four or more supplements [1].

Within the European Union, supplements are regulated as foods. A dedicated Directive (2002/24/EC) establishes common rules for products containing vitamins and minerals, including a list of permitted nutrients and authorized sources, together with labeling provisions; scientific evaluation of new micronutrient sources is conducted by the European Food Safety Authority (EFSA) under codified guidance and procedures [2–5]. Several key aspects remain only partially harmonized at EU level—most notably maximum/minimum levels for vitamins and minerals and the use of non-vitamin/mineral substances such as botanicals—so Member States are able to set national conditions of use, resulting in heterogeneous market access and oversight across the region [3, 6, 7]. In Italy, consumer surveys indicate high uptake of dietary supplements (DSs), estimating that about 70% of the adult population used supplements at least once in the previous year, and pharmacies remain the predominant purchasing channel [8, 9]. Complementing utilization figures, Italy has operated a national phyto-vigilance system since 2002; as of February 2025, more than 2,700 spontaneous reports of suspected adverse events (AEs) following the use of natural health products and DSs have been reported through the official website, www.vigierbe.it, supporting signal detection and regulatory communication [10–12].

Patients with chronic liver disease (CLD), including metabolic dysfunction-associated steatotic liver disease (MASLD), require particular caution when considering herbal and dietary supplements (HDS), given that limited hepatic reserve and polypharmacy could increase the risk of hepatotoxicity and drug interactions [13]. The current guidelines of the American and European Associations for the Study of the Liver, AASLD and EASL, respectively, underline that DSs are an increasingly frequent cause of liver damage; the recommendations indicate to routinely perform a well-structured anamnesis, specifically investigating the consumption of supplements, the use of standardized tools for determining causality and the possible recall of suspect products, after reporting them to the pharmacovigilance network [14, 15]. Furthermore, available evidence supports the relevance of the problem: a systematic review has mapped several herbal products associated with hepatotoxicity, documenting clinically serious outcomes, while through the RUCAM, more than 12,000 cases of herbal-induced liver injury (HILI) have been collected, highlighting the extent of the implicated products and the different degrees of causality [16, 17]. Despite this, herbal products based on silymarin and curcumin are widely marketed and studied as adjunctive therapies for MASLD [18–20], while multivitamins containing iron, folate, and vitamin B₁₂ are commonly used to prevent or correct deficiency anemia in at-risk adults [21, 22].

Thus, stemming from the analysis of this data, which justifies a targeted assessment of HDS exposure in CLD populations and supports the need not only for clear patient education but also for awareness among physicians, we conducted a cross-sectional survey to characterize HDS, oligo and trace element consumption among individuals with CLD, to examine sociodemographic correlates, to determine the extent to which use is overseen by general practitioners or specialists versus self-medication, and to document the most frequently reported reasons for use and perceived benefits.

## Methods

### Study population

The study was conducted at the chronic liver disease outpatient clinic of the Internal Medicine Unit and Hepatology of the University Hospital “P. Giaccone” of Palermo. Over a 6-month period (September 2023 to May 2024), patients with CLD and their caregivers were recruited consecutively and surveyed about their use of herbal, dietary supplements, oligo and trace elements. The questionnaire investigated demographic data, liver disease, lifestyle, use of supplements and/or herbal products, and information about them. All patients provided written informed consent, and the study was conducted according to the Declaration of Helsinki.

### Data collection

At the conclusion of each clinic visit, healthcare staff (doctors and nurses) administered a structured questionnaire to each patient. The questionnaire captured key demographic information, including age class (< 30 years, 30–40 years, 50–60 years, 60–70 years, > 70 years), gender, residential area (urban area, rural area), level of education (no qualification, primary school, middle school, high school diploma or bachelor’s degree), smoking habit (never, smoker, ex-smoker), alcohol use (absent, occasional, previous, present and if present specifying whether alcohol or spirits and number of alcoholic units/day), healthy lifestyle (diet, exercise, hours of sleep), use of supplements and/or herbal products (current/previous use, never used), reason for taking these substances (diabetes mellitus, dyslipidemia, weight loss, liver steatosis, varicose veins, menopause status, psychiatric disorders, anxiety and sleep disorders, urological disorders, headache, constipation or dyspepsia, electrolyte imbalances, osteoporosis, osteo-articular pain, flu syndromes, strengthening of immune system, pregnancy, infertility, personal well-being, improvement of sexual performance, or other); information on the product (type of supplement, method of intake, administration time, adverse effects, perceived benefits) and information regarding the prescription method and prescriber (nutritionist/dietician, general practitioner, specialist, pharmacist, herbalist, naturopath, internet, self-medication), along with whether the general practitioner was aware of the supplement use. It was not mandatory to complete all sections of the form, and the same questionnaire was given to caregivers. Data from these questionnaires were entered into a computerized database for analysis.

### Statistical analysis

Statistical analyses were performed using IBM SPSS Statistics, version 30 (IBM Corp., Armonk, NY, USA). Categorical variables were expressed as absolute numbers and percentages, while continuous variables were reported as means ± standard deviation. Differences between frequencies were evaluated using the Chi-square test, Fisher’s exact test, when appropriate. A two-tailed p value of < 0.05 was considered statistically significant.

## Results

Population of the study included 323 participants, 186 females (57.5%) and 137 males (42.5%); patients were 220 (68.1%) (116 F: 104 M) and caregivers 103 (31.9%), (70 F: 33 M); results are summarized in Table 1. Most patients belong to the older age class, with 61.4% aged more than 60 years; 22.3% are 40–65 years, while only 16.4% are less than 40 years. Comparing the age distribution, the group aged more than 65 years was significantly higher than that of caregivers (*p* = 0.001). However, there was no difference between the other age classes of the two study groups. The population also exhibits differences in sex distribution. Among caregivers, females account for 68.0%, whereas males represent 32.0%. Among patients, females 52.7% and males constitute 47.3%. Overall, this pattern indicates a higher female representation in caregiving roles, and the difference is statistically significant (*p* = 0.01).

**Table 1:** Clinical and demographic features of 323 subjects included in the survey

	Overall n = 323	Caregivers n = 103	Patients n = 220	*p*
Age (n, %)				
< 40 yrs	64 (19)	28 (27.2)	36 (16.4)	ns
40–65	78 (24)	29 (28.2)	49 (22.3)	ns
> 65	181 (56)	46 (44.6)	135 (61.4)	0.001
Sex (n, %)				
Female	186 (57.5)	70 (68.0)	116 (52.7)	0.001 *
Male	137(42.5)	33 (32.0)	104 (47.3)	
Residence area (n, %)				
Urban	281 (86,9)	85 (82.5)	196 (89.1)	ns
Rural	42 (13.1)	18 (17.5)	24 (10.9)	
Education (n, %)				
Primary school or less	207(64)	62 (60.2)	145 (65.9)	ns
High school/bachelor	116(36)	41 (39.8)	75 (34.1)	
Smoking (n, %)				
No	167 (51.7)	61 (59.3)	106 (48.2)	ns
Yes	156 (48.3)	42 (40.7)	114 (51.8)	
Alcohol (n, %)				
No	253(78.3)	86 (83.5)	167 (75.9)	ns
Yes	70 (21.7)	17 (16.5)	53 (24.1)	
Lifestyle (n, %)				
Healthy	224 (69.7)	77 (74.8)	147 (66,8)	ns
Unhealthy	99 (30.6)	26 (25.2)	73 (33,2)	
Liver disease referral (n, %)				
HBV	53 (24,1)	/	53 (24.1)	ns
HCV	48 (21.8)	/	48 (21.8)	
MASLD	82 (36.8)	/	82 (36.8)	
Miscellanea	37(16,8)	/	37(16,8)	
Supplements user (n, %)				
No	213 (65,9)	64 (62.1)	149 (67,7)	ns
Yes	110 (34.1)	39 (37.9)	71 (32,3)	

We assessed educational level and residency. Regarding educational level, there were no significant differences between the two study groups. About residency, most of the population resides in urban areas (86.9%), whereas a smaller proportion comes from rural areas (13.1%). Among patients, 89.1% live in urban settings and 10.9% live in rural areas, suggesting that most patients access care in the cities.

The population exhibits distinct lifestyle patterns across alcohol use, smoking, and health-conscious behaviors. Overall, alcohol consumption is uncommon (21.7%), with a higher prevalence among patients than caregivers (24.1% vs 16.5%). A large majority report a healthy lifestyle (69.7%), with slightly greater adherence among caregivers (74.8% vs 66.8%). Current smoking is reported by 48.3% of participants overall and is more frequent in patients than in caregivers (51.8% vs 40.7%).

The etiology of CLDs was distributed as MASLD (36.8%), HBV (24.1%), HCV (21.8%) followed by miscellanea (16.8%), which included a small percentage of autoimmune liver disease (2.3%), as well.

**Fig. 1:**
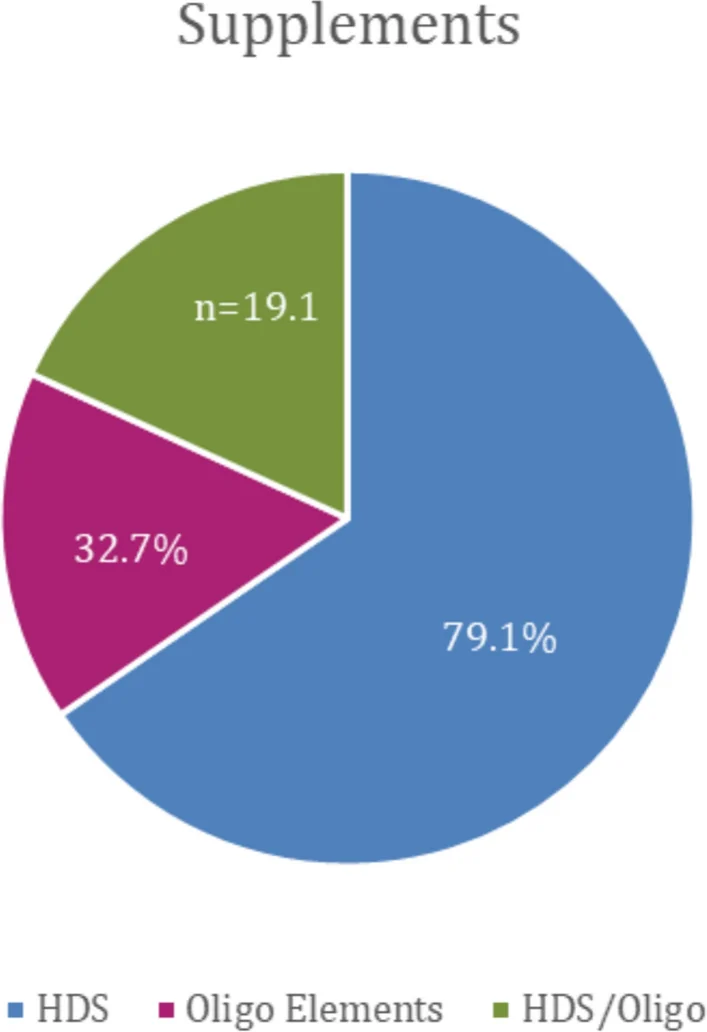
Class of HDS, oligo and trace element consumption among patients and caregivers

Most of the participants reported no supplement use (66.0%), and only 34.0% used at least one product. Thus, consumption of supplements was common for an overall of 110 participants out of 323 (34%), whereas patients were 71 (32.3%) and caregivers were 39 (37.9%). In Fig. 1, class of supplements’ consumption has been shown. Among users, herbal and dietary supplements were the most common (79%), followed by oligo and trace elements (33%); 19% reported using both, with no differences between patients and caregivers. Among users, perceived clinical benefit was reported by 70.9% overall (79.5% caregivers and 66.2% patients); adverse effects were uncommon (3.6%) (Table 2).

**Table 2:** Awareness of GP and consumption of HDS, oligo and trace elements among the 110 supplement consumers

	Overall n = 110	Caregivers n = 39	Patients n = 71	*p*
Awareness (n, %)				
Yes	92 (83.6%)	31 (79.5%)	61 (85.9%)	ns
No	18 (16.4%)	8 (20.5%)	10 (14.1%)	
Side effects (n, %)				
No	106 (96.4)	36 (92.3)	70 (98.6)	ns
Yes	4 (3.6)	3 (7.7)	1 (1,4)	
Clinical benefits (n, %)				
No	32 (29.1)	8 (20.5)	24 (33.8)	ns
Yes	78 (70.9)	31 (79.5)	47 (66.2)	
HDS (n, %)	87 (79.1)	30 (76.9)	57 (80.3)	ns
Oligo elements (n, %)	36 (32.7)	13 (33.3)	23 (32.4)	ns
HDS/oligo (n, %)	21 (19.1)	5 (12.8)	16 (22.5)	ns

*HDS* herbal and dietary supplements

Common reasons for HDS consumption varied among participants (Table 3). The most frequently reported motivations were anxiety (13.6%), sleep disorders (13.6%), and osteoporosis/osteoarthrosis (12.7%). Anxiety was significantly more frequent among patients than caregivers (12.7 vs 0.9%, *p* = 0.017), pointing to a psychosocial component that may drive supplement use in individuals with CLD, whereas sleep disorders and osteoporosis/osteoarthrosis did not differ by group. Anemia was the common cause of iron intake in 8/71 (7.3%) of patients as compared with 0/39 in caregivers (*p* = 0.049). Similarly, supplement intake was more frequent in patients with steatosis, 8/71 (7.3%) vs caregivers 0/39 (*p* = 0.049). The next most cited reasons included osteoporosis/osteoarthrosis (13,6%), dyslipidemia (10.9%), dyspepsia (10.0%), and electrolyte disorders (9.1%). These were followed by bowel disorders (8.2%), varicose veins (7.3%), liver steatosis (7.3%), weight loss (7.3%), and “well-being” (7.3%); none of these differed significantly between patients and caregivers. Rare motivations were limited to diabetes mellitus (2.7%); no entries were recorded for “flu syndromes,” infertility, or sport-related purposes.

**Table 3:** Common reasons for HDS, oligo and trace element consumption among the 110 participants

	Overall n = 110(%)	Caregivers n = 39(%)	Patients n = 71(%)	*p*
Anemia	8 (7.3)	0 (0.0)	8 (11.3)	0.049*
Anxiety	15 (13.6)	1 (0.9)	14 (12.7)	0.017*
Asthenia	10 (9.1)	4 (3.6)	6 (5.5)	ns
Well-being	8 (7.3)	4 (3.6)	4 (3.6)	ns
Diabetes	3 (2.7)	0 (0.0)	3 (2.7)	ns
Headache	4 (3.6)	1 (0.9)	3 (2.7)	ns
Weight loss	8 (7.3)	3 (2.7)	5 (4.5)	ns
Dyslipidemia	12 (10.9)	3 (2.7)	9 (8.2)	ns
Electrolyte disorders	10 (9.1)	5 (4.5)	5 (4.5)	ns
Dyspepsia	11 (10.0)	2 (1.8)	9 (8.2)	ns
Sleep disorders	15 (13.6)	2 (1.8)	13 (11.8)	ns
Bowel disorders	9 (8.2)	4 (3.6)	5 (4.5)	ns
Osteoarthrosis	15 (13.6)	5(4.5)	10 (9.1)	ns
Menopause	7 (6.4)	2 (1.8)	5 (4.5)	ns
Urological disorders	7 (6.4)	2 (1.8)	5 (4.5)	ns
Immunity	7 (6.4)	2 (1.8)	5 (4.5)	ns
Liver steatosis	8 (7.3)	0 (0.0)	8 (11.3)	0.049*
Varicose veins	8 (7.3)	4 (3.6)	4 (3.6)	ns
Other	5 (4.5)	4 (3.6)	2 (1.8)	ns

^*^Fisher’s exact test

**Fig. 2:**
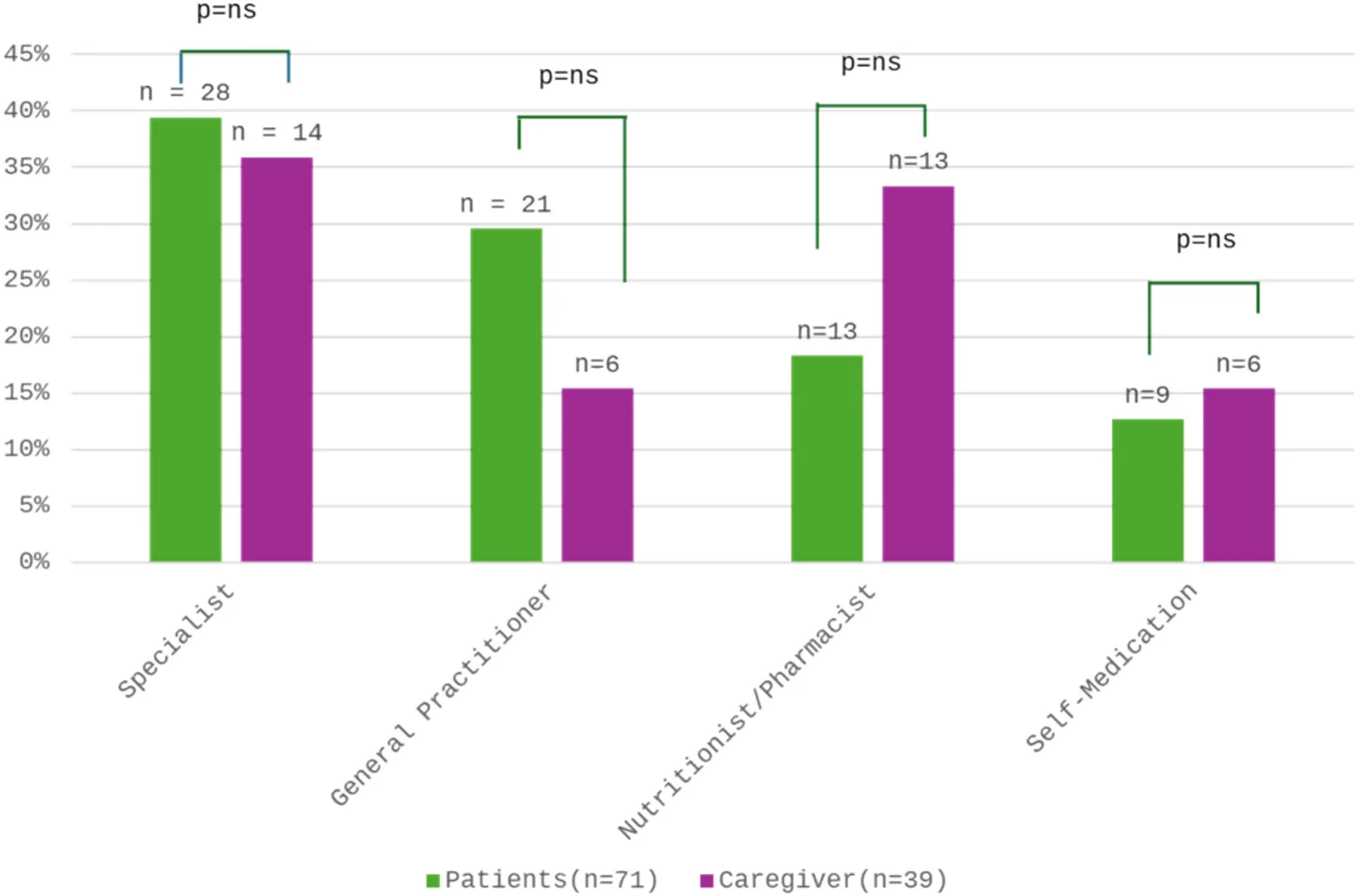
Source of prescription and awareness among patients and caregivers consuming HDS

Regarding prescriber awareness, as it has been shown in Fig. 2, the most frequently cited sources were specialists (13.0% of the entire sample), general practitioners (8.4%), and nutritionists/pharmacists (8.0%), while a minority reported self-medication/Internet (2.2%) or other sources (2.5%); no between-group differences emerged. When examining the sources of recommendations or prescriptions for supplements, clear patterns appear. Medical specialists were the most frequently cited source, reported by 39.4% of patients and 35.9% of caregivers. General practitioners were the next most common source among patients, 21 patients (29.6%) versus 6 caregivers (15.4%). Advice from nutritionists/pharmacists was reported equally in absolute numbers (13 caregivers; 13 patients), corresponding to 33.3% of caregivers and 18.3% of patients. Self-medication/Internet was less common overall (6 caregivers, 15.4%; 9 patients, 12.7%).

## Correlation between supplement consumption, gender, and age classes

Considering gender, supplement use for liver steatosis was more common among males (6/42; 14.3%) than females (2/68; 2.9%; *p* = 0.05). Stratifying by age class, a significant association between patient status and supplement use emerged only in participants younger than 40 years: supplements were used by 17/36 (47.2%) of patients versus 6/28 (21.4%) of caregivers (*p* = 0.039). No significant differences in supplement use between patients and caregivers were observed in the less-than-60 or more-than-65 age groups class, nor in the overall sample. Across age classes, osteoporosis/osteoarthrosis-related supplement use was significantly more frequent in participants 13/61 aged more than 65 years compared with those younger, 2/49 (21.3% vs 4.1%, *p* = 0.011).

## Discussion

As the use of herbal and dietary supplements (HDS) in patients with chronic liver disease is increasingly widespread, we decided to carry on this cross-sectional study to assess HDS, oligo and trace element consumption, to examine sociodemographic correlates, to determine the extent to which use is overseen by general practitioners or specialists versus self-medication, and to document reasons for use and perceived benefits.

From a demographical point of view, within our cohort of 323 participants, most patients belong to the older age class (61.4%), whereas 22.3% are aged between 40 and 65 years, and only 16.4% are less than 40 years. This distribution is consistent with the greater clinical burden typically observed in older adults accessing care. In this regard, caregivers show a more balanced age profile, indicating that caregiving responsibilities are shared across generations, with a substantial contribution from middle-aged adults and a meaningful involvement of older individuals, likely assisting spouses or other relatives.

In our study, 110 (34%) out of 323 participants reported using at least one HDS or oligo/trace element. Consumption was more frequent among caregivers than patients (37.9% vs 32.3%), indicating that individuals not directly affected by chronic conditions might be more inclined toward preventive supplementation or might have different health perceptions regarding supplement benefits [23]. Among users, HDS were the predominant choice, followed by oligo/trace elements; nearly 19% combined both categories. The predominance of HDS likely reflects the perception that “natural” products are safer or better tolerated; however, in the setting of CLD, this assumption warrants caution. Clinical Practice Guidelines from AASLD and EASL assessed the herb-induced liver injury (HILI) across a wide range of botanicals and multi-ingredient formulations, and recommended systematic history-taking, reconciliation of non-prescription products, use of standardized causality tools, as RUCAM, and prompt withdrawal of suspected agents [14–17]. In Italy, National phyto-vigilance resources support routine monitoring and reporting of suspected adverse reactions to natural health products through the official website [24, 25].

Regarding awareness of supplement use, nearly 62.7% of users received advice from doctors, specialists, or primary care physicians, and a further 23.6% consulted nutritionists or pharmacists. However, 6.4% initiated supplement use independently (self-medication/Internet), and another 7.3% cited non-medical sources. Overall, these data indicate that specialist consultations are predominant, while primary care and pharmaceutical/nutritional channels play a significant role in initiating supplement use. Another issue to consider, no less important than the recommendation, is the frequency with which primary care physicians are informed about supplement use. In our study, the majority of patients (83.6%) informed their primary care physician. This reflects a fairly good level of communication, facilitating monitoring for interactions and AEs. However, the fact that approximately 13.7% of the individuals involved started taking supplements completely independently (as self-medication/non-medical source) introduces a significant problem. Indeed, in the context of liver disease patients requiring polypharmacy, unsupervised use underscores the importance of structured and routine supplement screening and medication reconciliation during follow-up visits. Finally, the use of trace elements (e.g., iron, magnesium, zinc, selenium) is commonly associated with self-care goals (asthenia, fatigue, immune support), while targeted correction of documented deficiencies, such as iron for iron-deficiency anemia, should be clinically supervised to avoid AEs or ADRs [26, 27].

In general, supplement use appeared to be driven by both preventive intentions and symptom management. As compared with caregivers, in our study, patients more often reported anxiety, anemia, and liver steatosis as reasons for intake, consistent with a predominantly symptom- or diagnosis-driven pattern of HDS use within the CLD group. Steatosis-related intake clustered around hepatoprotective and antioxidant products (e.g., milk thistle, turmeric/curcumin, vitamin E), mirroring marketing claims and emerging trial evidence that silymarin-, curcumin- and vitamin E-based regimens can improve biochemical or histological features of NAFLD [18, 28–31]. In this regard, use of supplements specifically for liver steatosis was reported more frequently by men as compared with women (14.3% vs 2.9%; *p* = 0.05). Steatosis-related use mainly involved products *such* as milk thistle, turmeric/curcumin, vitamin E, omega-3 fatty acids, red yeast rice, belonging to the categories of “liver and detox/antioxidants” and “lipid-lowering nutraceuticals”. This consumption is broadly consistent with the higher prevalence of MASLD reported in men at reproductive *ages* and in women at early post-menopausal ages and with growing, though heterogeneous, evidence that silymarin, vitamin E, and omega-3-based regimens can improve steatotic liver disease [32–36]. However, it is noteworthy that among patients with steatotic liver disease lower than 40 years, supplement consumption appears more frequent among women with higher educational attainment [37]. Anemia was typically managed with multivitamin preparations, in line with the central contribution of combined micronutrient deficiencies to anemia in older adults [38, 39]. This may partly reflect differences in nutritional status and diet quality, as older adults living alone are at increased risk of malnutrition, all of which are relevant to anemia and non-specific symptoms and may prompt the use of multivitamin preparations and “neurotonic” products [40–42]. Osteoporosis/osteoarthrosis-related supplement use showed a clear age gradient, being rare below 65 and peaking in those more than 60, with a significant positive correlation with age (*p* = 0.011), echoing guidance to prioritize calcium/vitamin D supplementation in older adults at risk of osteoporosis or with low dietary intake [43].

In our study among HDS users, perceived clinical benefit clearly outweighed harm: about 70.9% of users reported symptomatic improvement, more frequently in caregivers than in patients, whereas AEs were uncommon (3,6%). This pattern is in line with population surveys of HDS users, whereas most consumers describe these products as helpful and only a minority report suspected AEs. A recent review of 141 clinical trials, evaluating the outcomes of herbal product use for common indications, supports the notion that HDS are generally well tolerated, do not cause serious adverse events, and in some clinical settings, the benefits may often outweigh the risks [44]. A similar, real-world study of herbal medicinal products used for gynecological disorders suggests a favorable benefit–risk ratio with only 1.9% adverse events [45]. At the same time, data from pharmacovigilance systems and liver injury registries show that HDS may explain a portion of DILI, suggesting that serious toxicity is likely underestimated in self-report surveys. Nonetheless, the cross-sectional, self-report design of our study almost certainly underestimates harms and precludes causal attribution for both benefits and AEs [46–49]. Despite the generally positive attitude toward supplements due to compliance with EU regulations, the potential risks associated with their use cannot be overlooked. HDS, although popular, have been linked to adverse liver events, a concern initially highlighted by Goldman and further discussed by Licata et al. [50, 51]. Additionally, ADRs and organ damage have been noted in the literature, underscoring the need for careful monitoring of long-term supplement use [51, 52]. However, the absence of serious AEs could stem from our population’s adherence to EU regulations, which strictly govern the dietary supplement market, making them relatively safe even for patients with liver disease. In Italy, a website is available for reporting AEs and ADRs related to dietary supplements, www.vigierbe.it. Although there are adequate rules and an official website, the “underreporting” of AEs, due to the underuse of these tools, as usually happen per conventional drugs, represents still a relevant problem.

Finally, in our study, we found a significant association between CLD and the use of supplements for anxiety, suggesting that emotional distress may play a role in driving self-medication. While most users perceived benefits from supplementation, caregivers, who did not suffer from CLD, reported a greater perceived benefit than patients. This difference may reflect the psychological burden of chronic disease on expectations and responses to treatment [53]. In this regard, the higher percentage of anxiety in patients with CLD is related to the chronic disease state itself and the resulting uncertainty, which is precisely why patients often turn to alternative medicines. This gives rise to reflection on the complexity of managing a patient with chronic disease, and particularly the management of a patient with cirrhosis, for which the approach should be “patient-centered” rather than “disease-centered” [54].

This study has some limitations. Its cross-sectional nature and single tertiary referral center limit causal inference and generalizability to other settings and less selected CLD populations. Supplement use was assessed by self-report with a non-validated questionnaire, without systematic verification of brands, doses or duration, making recall and misclassification bias possible. Finally, caregivers—who in our sample reported more frequent use of HDS than patients—were not characterized in comparable clinical detail, so this finding should be interpreted with caution and confirmed in specifically designed studies.

In conclusion, due to the lack of standardized regulation and the underreporting of dietary supplement consumption, this survey highlights that ensuring appropriate, safe, and consistent use of HDS requires a multidisciplinary approach involving internists, hepatologists, and nutritionists. In the context of an aging population suffering from chronic disease, caregivers play a key role, acting as intermediaries between the patient and the healthcare system. Healthcare systems, social services, and public authorities must ensure that vulnerable patients are not left alone to manage complex healthcare decisions, especially when it comes to taking potentially harmful over-the-counter products, such as dietary supplements. Protecting these patients requires not only clinical vigilance, but also collective social commitment to promote informed and safe use of supplements.

## Supplementary Information

Below is the link to the electronic supplementary material.

Supplementary file1 (DOCX 14 KB)

## Data Availability

The datasets generated during and/or analysed during the current study are available from the corresponding author on reasonable request.
