# Supplementary material for: Herbal, dietary supplements, oligo and trace elements consumption among chronic liver disease patients and caregivers: a cross-sectional study
Source: Intern Emerg Med. 2026 Jun 23;21(7):2557–65. doi: 10.1007/s11739-026-04425-8 (PMC13694334; doi:10.1007/s11739-026-04425-8)
Supplement: Supplementary file 1 [file 11739_2026_4425_MOESM1_ESM.docx]

**Supplementary Table 1. Overview of reported supplements (grouped by category)**

**Minerals & trace elements:** magnesium, potassium, manganese.

**Vitamins&multivitamins:**vitamin D, vitamin C, vitamin E, vitamin K, B-complex, multivitamins.

**Amino acids, proteins& performance:** arginine, L-arginine, creatine, proteins, L-acetyl-carnitine, GPE, glutathione, inositol, omega-3.

**Fibers&laxatives /GI:**macrogol, psyllium, bran, acid-control products.

**Lipid-lowering nutraceuticals:** red yeast rice–based products, berberine, coenzyme Q10; additional brand formulations.

**Urological/andrological:**Serenoa repens, D-mannose and cranberry, male fertility products, arginine-based products.

**Sleep & calming /neurotonics:** melatonin, valerian, calming blends,neurotropic complexes

**Vascular & microcirculation:**oxerutin/rutosides, diosmin-hesperidin, additional venotonic formulations.

**Joint/bone/connective tissue:** collagen, chondro-protectives, joint mixes, calcium/vitamin D adjuncts.

**Liver & detox / antioxidants:** milk thistle (cardo mariano), hepatoprotective/antioxidant combos, turmeric (curcuma), Chelidonium.

**Immune support & botanicals:** quercetin, blueberry/cranberry, herbal 10-herb blends, ribes&betulla, malva, chamomile, passiflora.

**Medical devices/unclear brands :** Protocol, Kuzik, Trixy, Triscudil, Faros, other brand-only entries.

**Other unclear :** entries with insufficient detail for classification.

**Supplementary Table 2. Survey on Dietary Supplements and Herbal Products**

*Indicates a mandatory question*

**Patient / Caregiver**

**1. Patient’s age ***

Select one option.

- < 30 years
- 30–40 years
- 40–50 years
- 50–60 years
- 60–70 years
- > 70 years

**2. Gender ***

Select one option.

- Male
- Female

**3. Place of residence ***

Select one option.

- Urban area
- Rural area

**4. Marital status ***

Select one option.

- Single
- Married
- Separated / Widowed

**5. Educational level ***

Select one option.

- No formal education
- Primary school certificate
- Middle school diploma
- High school diploma
- University degree

**6. Cigarette smoking ***

Select one option.

- Never
- Smoker
- Former smoker

**7. Alcohol consumption ***

Select one option.

- No
- In the past
- Occasionally
- Yes

If yes, specify type (alcoholic / super‑alcoholic / both):
_____________________
If yes, specify number of drinks per day:
_____________________

**8. Healthy lifestyle habits ***

Do you believe you have healthy lifestyle habits (diet, sleep duration, etc.)?
Select one option.

- Yes
- No

**9. Use of supplements/herbal products ***

Select one option.

- No
- Yes
- In the past

**10. Reason for use ***

Select one option.

- Arterial hypertension
- Diabetes mellitus
- Dyslipidemia
- Weight loss
- Hepatic steatosis
- Varicose veins
- Menopause
- Anxiety and/or sleep disorders
- Psychiatric disorders
- Urological conditions (cystitis, prostatic hypertrophy, etc.)
- Headache
- Constipation and/or dyspepsia
- Electrolyte disturbances
- Osteoporosis
- Joint pain
- Products for flu-like conditions
- Products to strengthen immune system
- Pregnancy
- Fertility enhancement
- Sports performance products
- Well-being / personal wellness
- Products to improve sexual performance
- Other (specify): _____________________

**11. Product details**

Specify product type, dose, and duration:
_____________________
_____________________
_____________________

**12. Side effects ***

Select one option.

- No
- Yes (specify): _____________________

**13. Perceived benefit ***

Select one option.

- No
- Yes (specify): _____________________

**14. Who recommended the product? ***

Select one option.

- Nutritionist / Dietitian
- General Practitioner
- Specialist Physician
- Pharmacist
- Other (Herbalist, Naturopath, etc.)
- Mass media / Internet
- Self-medication

**15. Is the General Practitioner aware? ***

Select one option.
- Yes
- No
